# Exploring the Relationships between Structure and Antimicrobial Potency of Quinolinequinones

**DOI:** 10.3390/antibiotics11101397

**Published:** 2022-10-12

**Authors:** Emel Mataracı-Kara, Nilüfer Bayrak, Mahmut Yıldız, Hatice Yıldırım, Amaç Fatih TuYuN

**Affiliations:** 1 Pharmaceutical Microbiology Department, Pharmacy Faculty, Istanbul University, Beyazit, Istanbul 34116, Turkey; 2 Department of Chemistry, Faculty of Engineering, Istanbul University-Cerrahpasa, Avcilar, Istanbul 34320, Turkey; 3 Chemistry Department, Gebze Technical University, Gebze, Kocaeli 41400, Turkey; 4 Department of Chemistry, Faculty of Science, Istanbul University, Fatih, Istanbul 34126, Turkey

**Keywords:** quinolinequinones, antibacterial activity, antifungal activity, antibiofilm activity, kinetic study, bactericidal effect

## Abstract

Microorganisms are responsible for hospital infections, and methicillin-resistant *Staphylococcus aureus* is one of them. In looking for the most effective lead structures to cope with the rise of antimicrobial (antibiotic) resistance, we evaluated the antimicrobial profile of quinolinequinones for potential antimicrobial applications. 1,4-quinone molecules fused with heteroatom have been studied extensively for many years as a source of drugs and lead structures. The aims of this study were to evaluate the antimicrobial activity of quinolinequinones against bacterial and fungal strains, and to probe for potential lead structures. For this reason, the activity of these compounds against three different strains of *Candida* fungi (*C. albicans*, *C. parapsilosis*, and *C. tropicalis*) and Gram-positive and Gram-negative pathogenic bacteria were investigated, searching for potential lead compounds. Five of nine quinolinequinones showed activity mainly against the Gram-positive strains with a minimal inhibitory concentration within the Clinical and Laboratory Standards Institute (CLSI) levels. The results revealed that quinolinequinones have significant activity against bacteria including *Staphylococcus aureus* and *Staphylococcus epidermidis*, and fungi including *Candida albicans* and *Candida parapsilosis*. **QQ1**, **QQ2**, **QQ3**, **QQ5**, and **QQ6** exhibited the highest growth inhibition against two essential species of the Gram-positive strains (*Staphylococcus epidermidis* and *Staphylococcus aureus*). Among these, four molecules (**QQ2**, **QQ3**, **QQ5**, and **QQ6**) were also active against *Enterococcus faecalis*, the other member of the Gram-positive strains. The antifungal profile of two quinolinequinones (**QQ7** and **QQ8**) indicated that they were as effective as the reference drug Clotrimazole against *Candida albicans*. The same molecules also have potential inhibitory antifungal activity against *Candida tropicalis*. For better understanding, the most active two quinolinequinones (**QQ2** and **QQ6**) were examined for biofilm inhibition and a time-kill kinetic study.

## 1. Introduction

Nature is a crucial source of drugs with an ample supply of versatile active pharmaceutical ingredients (APIs), obtained from natural sources [1]. An impressive number of APIs, especially antibiotics used in human medicine, are natural molecules produced by microorganisms, showing the ability to inhibit the growth of bacteria or fungi. However, bacterial resistance due to adaptations of microorganisms caused by overuse and/or abuse of antibiotics has appeared in a group of patients less than ten years after the appearance of the molecule in the market [2,3]. As an inexhaustible source of drug discovery, natural products especially defined as small molecule secondary metabolites are privileged structures to develop new antimicrobial lead compounds in response to the growing antibiotic resistance problem [4]. Bacteria produce antimicrobial substances that kill or inhibit the replication of other antimicrobials. In other words, scientists can search for new antibiotics wherever bacteria originate, even in the human body. Indeed, the isolation of API from natural sources, especially from *Streptomyces*, has brought with many drugs currently used in fighting the treatment of various diseases [5,6]. Streptomycin, a marketed antibiotic, has been derived from bacteria that grow on artificial solid or liquid media [7]. Daunorubicin and doxorubicin, a hydroxylated analog of daunorubicin, are the most prominent members isolated from diverse *Streptomyces* strains [8].

Nowadays, tremendous efforts are required to cope with emerging antimicrobial resistance that constantly infects the human population and threatens global public health. Since most drugs commercially available in the market or approved worldwide are related to a natural product origin, natural products have had a majority as templates or direct impacts of treatment in health care [9]. Despite the growing demand for new therapeutic compounds from natural sources, their isolation, scale-up, and structure elucidation remain intensive processes [10,11]. Thus, a rational and hopeful strategy targeting microorganisms such as bacteria and/or fungi with molecules from natural sources is required. As an alternative, scientists in medicinal chemistry and biological chemistry generally use a concept, namely, the natural products derivatization method, as a method of drug development by modifying the pharmacophore of natural products to construct libraries of natural product analogs and natural product-like compounds [12].

Quinone compounds are a kind of cyclic molecule with two ketone moieties widely found in natural products with extensive biological activity in biology and medicine. For example, thymoquinone is the vital component of *Nigel sativa* L. seeds [13], showing high antibacterial activity against methicillin-resistant *Staphylococcus aureus* (MRSA) [14] and *H. influenzae* [15] and moderate activity against *S. pneumoniae* [15]. The promising quinones from nature are ubiquinones and plastoquinones, responsible for the electron transfer chain in plant cells during photosynthesis in plant growth and development [16]. The literature shows that the heterocyclic quinones containing one or more heteroatoms, especially nitrogen [17], called azaquinones, represent one of the most abundant derivatives of quinone molecules [18]. Azaquinones are an important family with diverse biological activities [18,19]. The azaquinone moiety is known to afford antimicrobial and/or anticancer activity in a number of clinically used streptonigrin [20] or mitomycins drugs [21]. In consideration of the importance of azaquinones, we recently synthesized a series of quinolinequinones containing aminophenyl rings with different substituents via a previously reported two-step route [22] and evaluated their in vitro cytotoxic activity against four different cancer cell lines (K562, Jurkat, MT-2, and HeLa) and human peripheral blood mononuclear cells (PBMCs) by MTT assay with further mechanistic studies assisting in understanding the mainly apoptotic effects of these analogs in addition to the ABL1 kinase inhibitory activity and DNA cleavage activity [22]. In this study, the activity and mode of action of these compounds against seven different pathogenic bacterial and three fungal strains were investigated in order to determine the antibacterial and antifungal profile of quinolinequinones. In order to provide greater insight into the chemical features that might enhance antimicrobial activity, the aminophenyl ring was substituted with a variety of chemically diverse but pharmacologically important groups in different positions. Towards this direction, a bactericidal time-kill kinetic study, antibiofilm activity, and potential antimicrobial activity (20 different clinically obtained strains) were investigated. Structure–activity relationship models (SAR) were generated in an effort to provide a putative explanation of the correlation among key parameters of the quinolinequinones that might influence their activity.

## 2. Materials and Methods 

### 2.1. Chemistry

Synthesis of the quinolinequinones (**QQ1**–**9**) has been reported previously, and references are cited therein [22].

### 2.2. In Vitro Antimicrobial Activity

#### 2.2.1. Determination of Minimum Inhibitory Concentrations (MICs)

Antimicrobial studies were determined by the broth microdilution technique using the Clinical Laboratory Standards Institute (CLSI) recommendations [23,24]. Mueller–Hinton broth (BD Difco, Detroit, MI, USA catalog number: DF0757-17-6) for bacteria ((*Staphylococcus aureus* (ATCC 29213), *Staphylococcus epidermidis* (ATCC 12228), *Enterococcus faecalis* (ATCC 29212), *Pseudomonas aeruginosa* (ATCC 27853), *Escherichia coli* (ATCC 25922), *Klebsiella pneumoniae* ATCC 4352), and *Proteus mirabilis* (ATCC 14153)), and RPMI-1640 medium buffered to pH 7.0 with MOPS (Sigma, St. Louis, MO, USA, R6504, M1254) for the yeast strains ((*Candida albicans* (ATCC 10231), *Candida tropicalis* (ATCC 750), and *Candida parapsilosis* (ATCC 22019)) were used as the test media. Then 10 mg/mL stock solution of each molecule was prepared using DMSO (Merck, Catalog number: 67685). Then, serial twofold dilutions ranging from 1250 µg/mL to 0.6 µg/mL were prepared in the medium. The inoculum was diluted in broth media to give a final concentration of 5 × 10^5^ CFU/mL for bacteria and 0.5 × 10^3^ to 2.5 × 10^3^ CFU/mL for yeast in the test tray. The MIC was defined as the lowest concentration of compound producing complete inhibition of visible growth [23,24]. The antimicrobial effects of the solvents were also investigated against the test microorganisms as controls, and the results were evaluated according to the values of the controls. According to the results, in vitro activities of **QQ2** and **QQ6** were investigated against each of the 31 resistant *Staphylococcus aureus* strains obtained clinically by the broth microdilution technique recommended in CLSI [23,24]. Thirty-one nonduplicates, nosocomially acquired methicillin-resistant *Staphylococcus* spp. isolated from blood specimens between June and December 2018, were obtained from the Department of Infectious Diseases and Clinical Microbiology, Faculty of Medicine, Istanbul Medipol University. API STAPH (bioM’erieux) was used to identify the studied clinical strains. Additionally, all the tested *Staphylococcus* spp. were selected by using oxacillin susceptibility to examine the methicillin-resistant isolates, approved by CLSI (MIC ≥ 4 µg/mL).

#### 2.2.2. Determination of Bactericidal Effects by Time-Kill Curve Kinetics (TKC)

Time-kill studies were performed according to NCCLS guidelines [25]. To investigate the synergistic bactericidal effects against clinically resistant *S. aureus* and *S. epidermidis* strains, the selected molecules (**QQ2** and **QQ6**) and levofloxacin (Sigma, catalog number: 28266), **QQ2** + levofloxacin, and **QQ6** + levofloxacin were combined at a concentration of 1 × MIC for each strain. According to NCCLS [25], after adding antimicrobials, the starting inoculum was 1 × 10^6^ to 5 × 10^6^ CFU/mL. The test tubes containing MHB with and without (growth control) antimicrobials in a final volume of 10 mL were incubated in a 37 °C calibrated shaking water bath (Nukleon, catalog number: UM-NCS30L; 180 rpm), and viability counts were performed at 0, 2, 4, 6, and 24 h intervals after inoculation by subculturing 0.1 mL serial dilutions onto TSA (BD Difco, catalog number: 236950) plates. All tests were performed in duplicate. Time-kill curves were determined by plotting mean colony counts (log_10_ CFU/mL) versus time. The lower limit of detection for time-kill assays was one log_10_ CFU/mL. Antimicrobial carry-over was controlled by the inhibition of colonial growth at the site of the initial streak according to NCCLS guidelines [25]. According to NCCLS [25], bactericidal activity was defined as a decrease of ≥3 log_10_ CFU/mL from the initial inoculum at 24 h. The results were interpreted by the effect of the combination in comparison with its more active constituent. Synergy and antagonism were defined as a two log_10_ decrease or increase in the viable count of the combination at 24 h compared to the more active of the two agents alone by using NCCLS criteria [25], respectively.

#### 2.2.3. Biofilm Attachment and Inhibition of Biofilm Formation Assays

Biofilm attachment and inhibition of biofilm formation assays were performed as per the previously described method with some modifications [26]. According to the previously reported method by Yıldız et al. and Mataracı Kara et al. [27] for biofilm attachment assays, an overnight culture of *S. epidermidis* ATCC 35984 and *S. aureus* ATCC 43300 were added to each well of a 96-well tissue culture microtiter plate (Greiner, catalog number: M0812) with 1/10 the MICs of **QQ2** and **QQ6** compounds, respectively. The plates were incubated for 1, 2, and 4 h for the studied isolates at 37 °C [26,27].

We also determined the inhibition of biofilm formation methods by using those previously reported by Yıldız et al. and Mataracı-Kara et al. [26,27]. According to their experiments [26,27] for inhibition of biofilm formation, TSB-glucose (BD Difco, catalog number: DF0370-17-3) for *S. epidermidis* and *S. aureus* were incubated at 37 °C, 24 h, with the tested QQ compounds at 1 × MIC, 1/10 the MIC, and 1/100 the MIC in 96-well tissue culture, respectively. Six wells were used for each molecule. The positive controls were microorganisms in TSB-glucose for *S. epidermidis* and *S. aureus* without molecules, respectively. After the incubation, wells were washed with PBS solutions and measured at OD_595_ nm (BioTek Eon Microplate Spectrophotometer).

### 2.3. Statistical Analysis

Two-way ANOVA-Tukey’s multiple comparison tests (Prism 8.0, GraphPad, San Diego, CA, USA) were used to compare differences between control and antimicrobial-treated biofilms, and experiments were performed using two independent assays. A *p*-value < 0.0001 was considered statistically significant.

## 3. Results

### 3.1. Chemistry

**Scheme 1:**
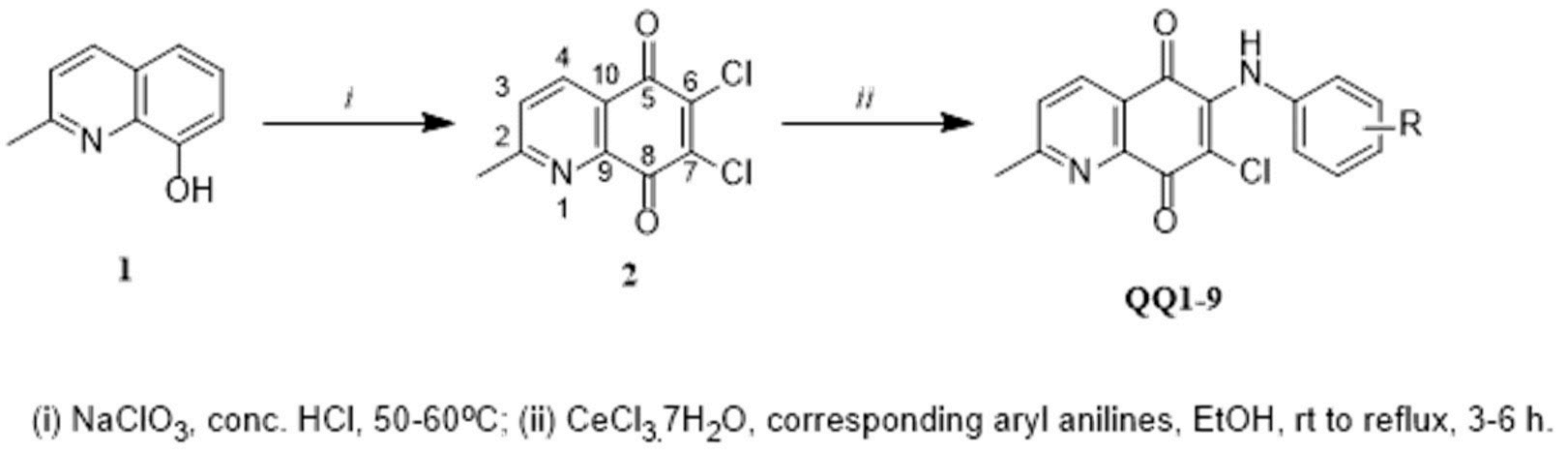
Preparation of the aminated QQs (**QQ1**–**9**).

The quinolinequinones used in the present study were prepared by the nucleophilic substitution reaction of the 6,7-dichloro-5,8-quinolinequinone synthesized by using Shaikh’s methodology starting from the commercially available 8-hydroxyquinoline (1) with sodium chlorate in concentrated HCl solution according to the procedure [20] with aryl amines in ethanol in the presence of CeCl_3_·7H_2_O as the catalyst, as shown in Scheme 1.

We found that the growth inhibition of dimethylquinone containing aryl amines having different substituents at various positions was substantially active [26]. We also designed and synthesized the quinolinequinones by linking the aryl amino group to azaquinone [22]. To develop improved antimicrobial molecules, we adopted a pharmacophore approach to merge the quinolinequinone and aryl amines containing an ester moiety. The present work extends our ongoing efforts toward developing biologically active molecules with high efficiency [26,27].

### 3.2. Library Screening

The genus *Staphylococcus*, one of the Gram-positive strains, is a member of the Micrococcaceae family from the phylum Actinobacteria. In addition to being resistant to most disinfecting agents, they can survive in harsh conditions, such as outside the body [28,29]. Regarding the development of effective antimicrobial agents, particularly against methicillin-resistant *Staphylococcus* spp. strains, quinolinequinones are classified as having the potential for developing novel antibacterial candidates [27,30,31]. Other noteworthy examples are sulfanyl and amino analogs of quinolinequinones, which play a significant role against MRSA, as published by Yildirim and Bayrak [32,33].

For these studies, each member of the library was studied for in vitro antimicrobial activity against a panel of Gram-positive bacteria and Gram-negative bacteria by using the broth microdilution technique to obtain the minimal inhibitory concentrations (MICs) according to the Clinical Laboratory Standards Institute (CLSI) recommendations [23,24]. The minimal inhibitory concentrations (MICs) determined against the standard strains of clinical importance used in this study were from the American Type Culture Collection (ATCC^®^) of the quinolinequinones (**QQ1**–**9**) and are summarized in Table 1 and Table 2.

Primarily, the quinolinequinones (**QQ1**–**9**) were evaluated for in vitro antibacterial profile detection against Gram-positive and Gram-negative bacteria. All quinolinequinones showed no acceptable inhibitory activity (with the MIC values at 625–1250 μg/mL) against four reference strains, namely, all Gram-negative bacteria. Interestingly, the activity profiles were mainly against the Gram-positive strains. The results showed that the Gram-positive strains were the most susceptible to the quinolinequinones. The screening showed that five quinolinequinones were active molecules (**QQ1**, **QQ2**, **QQ3**, **QQ5**, and **QQ6**) against two important species of the Gram-positive strains (*Staphylococcus aureus* and *Staphylococcus epidermidis*). Among these, four molecules (**QQ2**, **QQ3**, **QQ5**, and **QQ6**) were also active against *Enterococcus faecalis*, the other member of the Gram-positive strains (Table 1). Remarkably, three quinolinequinones (**QQ1**, **QQ5**, and **QQ6**) displayed the most significant antibacterial activity against *S. aureus* with a MIC value of 1.22 μg/mL, which was equal to the reference drug cefuroxime-Na. Among all the tested molecules, two quinolinequinones (**QQ2** and **QQ3**) gave low MIC values of 2.44 μg/mL against *S. aureus*. These results indicated that these two molecules had the potency to be further studied. Furthermore, the quinolinequinones (**QQ1**, **QQ2**, **QQ3**, **QQ5**, and **QQ6**) showed 8-fold inhibitory potency against *S. epidermidis* than cefuroxime with a MIC value of 1.22 μg/mL. Noticeably, **QQ6** exhibited the best (27-fold more potent) anti-*E. faecalis* activity with a MIC value of 4.88 μg/mL, which was superior to the standard drug amikacin (MIC = 128 μg/mL). On the other hand, **QQ3** was the second most active one of this series against *E. faecalis* (13-fold more potent than amikacin with a MIC value of 9.76 μg/mL). Another two quinolinequinones (**QQ2** and **QQ5**) gave the best activity against *E. faecalis* with MIC values of 19.53 μg/mL, which was 6-fold more potent when compared with the standard drug amikacin.

Regarding the antifungal activity, Table 2 revealed that most quinolinequinones exhibited an inversely inhibitory tendency to their antibacterial activity, though weaker than the antibacterial activity. Predominantly, two quinolinequinones (**QQ7** and **QQ8**) showed a profound effect against *C. albicans* with a MIC value of 4.88 μg/mL, equal to the reference drug clotrimazole [34]. In addition, when we investigated the inhibitory activity of the same quinolinequinones (**QQ7** and **QQ8**) against *C. parapsilosis* strains, **QQ7** and **QQ8** had acceptable activity compared with the reference drug amphotericin B, which had 5-fold and 10-fold less inhibitory potency, respectively.

**Figure 1:**
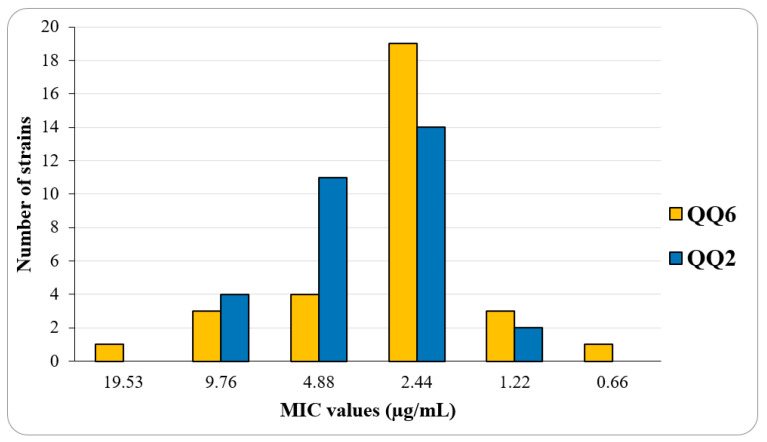
MIC distribution of **QQ2** and **QQ6** against 31 clinically obtained resistant strains of *Staphylococcus* spp.

Following our findings, we objected to specifying the potential antibacterial effects of **QQ2** and **QQ6**, which have prolonged activity analogs. They were selected for further analysis against clinically resistant isolates of *Staphylococcus* spp. isolates. Therefore, we explored their eventual antimicrobial activities against each of 31 clinically obtained resistant strains of *Staphylococcus* spp. The results indicated in Figure 1 from the MIC assessment showed that the MIC ranges for **QQ2** and **QQ6** were 1.22–9.76 and 0.66–19.53 μg/mL, respectively. In the present study, the in vitro antimicrobial activity of **QQ2** was up to 100% (MIC range 1.22–9.76 μg/mL) among all the studied isolates of clinically resistant *Staphylococcus* spp. and exceeded the susceptibilities to reference antimicrobial cefuroxime (MIC = 9.8 μg/mL). Overall, for the clinically resistant *Staphylococcus* spp. isolates, both **QQ2** (MIC_50/90_ = 2.44/9.76 μg/mL) and **QQ6** (MIC_50/90_ = 2.44/9.76 μg/mL) were the most active agents compared with reference antimicrobial cefuroxime.

### 3.3. Structure–Activity Relationships (SARs) Study for Biological Evaluation

**Figure 2:**
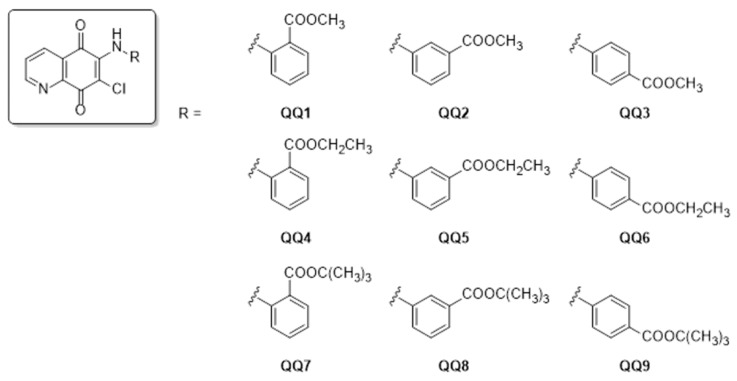
The structures of quinolinequinones with aryl amine containing different ester groups in different positions.

The SAR study was conducted to analyze the effect of the substituent with various positions [35]. The structures of the small library of the obtained quinolinequinones (**QQ1**–**9**) are shown in Figure 2. The SAR study demonstrated that the ester group, in particular unbranched alkyl chain (–COOCH_3_ and –COOCH_2_CH_3_) at all positions put forth a significant impact on biological activities for the antibacterial activity against *S. aureus*, *S. epidermidis*, and *E. faecalis* strains. Moreover, when the unbranched alkyl chain was replaced by a branched alkyl chain (COOC(CH_3_)_3_), the corresponding quinolinequinones (**QQ7**, **QQ8**, and **QQ9**) displayed weak or no prominent antibacterial activity. This fact pointed out that the branched alkyl chain was unfavorable for the antibacterial activity, and only a suitable length of the alkyl chain (methyl and ethyl) in the ester group was necessary for the antibacterial activity. The replacement of the ester group from ortho to meta or meta to para remarkably improved the antibacterial activity against *E. faecalis*.

The antifungal evaluation showed that most quinolinequinones, especially containing an ester group with an unbranched alkyl chain, showed weak to good bioactivity against most of the tested fungi. In contrast to antibacterial activity, two quinolinequinones (**QQ7** and **QQ8**) displayed better antifungal activity against *C. albicans* and *C. parapsilosis*.

In a previous study, our group demonstrated that the incorporation of dimethylquinone with aryl amines containing an ester group was not an efficient strategy to increase antibacterial and antifungal activity [26]. When aryl amines containing the ester group are coordinated with quinolinequinone, they induce structural changes, ultimately altering the molecule’s ability to interact with biomolecules, which may have a positive impact on the desired biological activity. Among nine active quinolinequinones against the bacteria and fungi, SAR analysis of the nine quinolinequinones tested indicated that the two quinolinequinones (**QQ2** and **QQ6**) presented remarkable activity against all Gram-positive bacteria, including *S. aureus*, *S. epidermidis*, and *E. faecalis*. Furthermore, these two quinolinequinones are responsible for the promising increase in antibacterial activity (Table 1). Thus, strikingly, they were selected for further studies to understand the mode of action, since they had a wide range of activity on the studied strains.

### 3.4. Bactericidal Effects by Time-Kill Curve Kinetic Study

**Figure 3:**
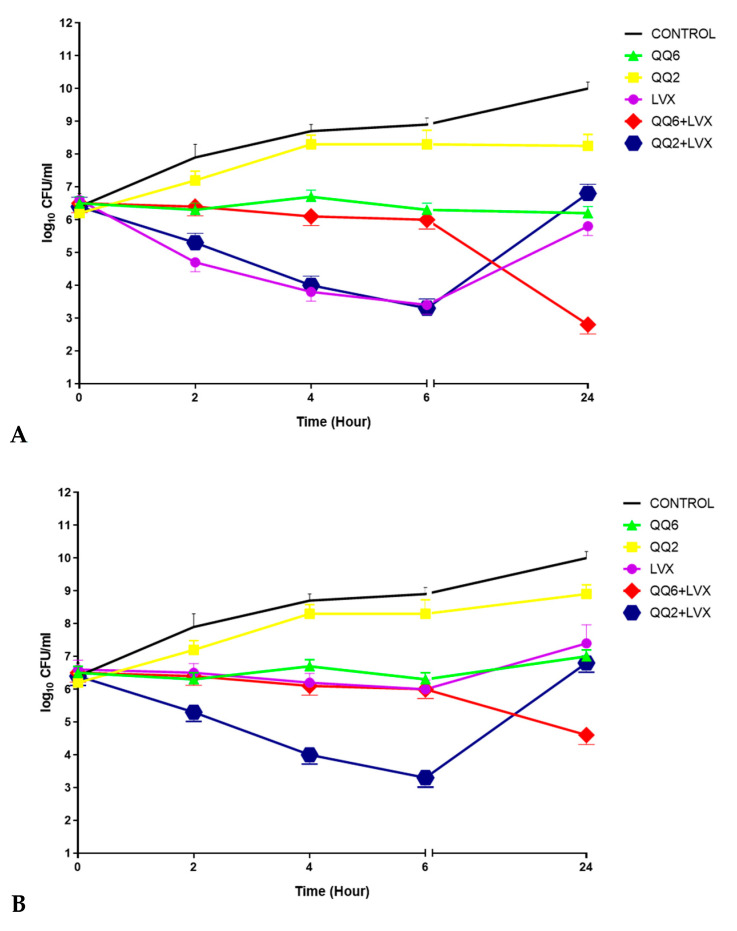
Time-kill determinations for clinically resistant (**A**) *S. aureus* and (**B**) *S. epidermidis* isolates after treatment with **QQ2** and **QQ6** alone or in combination with levofloxacin at 1 × MIC. The x-axis represents the killing time, and the y-axis represents the logarithmic *S. aureus* and *S. epidermidis* survival, respectively. Experiments were performed in duplicate.

The bactericidal activity of the most active two quinolinequinones (**QQ2** and **QQ6**) alone or in combination with levofloxacin was also examined from the library for further investigation using time-kill assays against clinically resistant *Staphylococcus aureus* and *Staphylococcus epidermidis* isolates at the respective MICs, and the results are given in Figure 3. Levofloxacin antibiotic, which shows high activity against Gram-positive bacteria containing *S. aureus*, has a fluoroquinolone structure [36]. Experimental studies have revealed that fluoroquinolones combined with standard antistaphylococcal therapy in severe *S. aureus* infections have an additive effect [37].

The results of the time-kill curve studies generally showed that neither **QQ2** nor **QQ6** displayed bactericidal activity (with a 3-log_10_ killing) in accordance with the 1 × MIC concentration used within 24 h. However, synergistic interaction was achieved in all strains studied with the combination of **QQ6** and levofloxacin used in 1 × MIC, and different antagonisms were not observed in different combinations.

### 3.5. Effect of Quinolinequinones on Biofilm Formation

**Figure 4:**
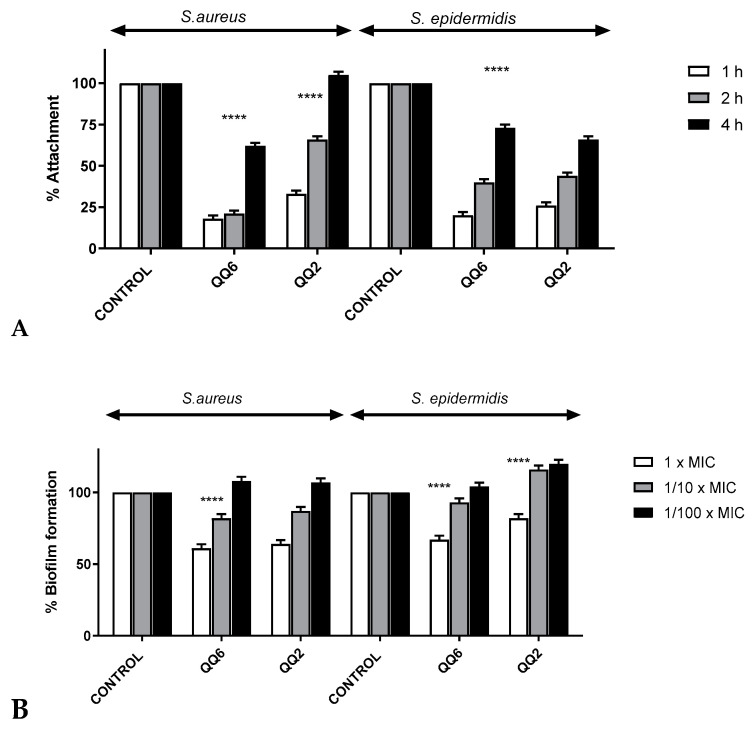
Inhibition of *S. aureus* and *S. epidermidis*. (**A**): surface attachment to the wells contained 1/10 × MIC of molecules and an inoculum of 1 × 10^6^ CFU/200 μL, incubated for 1, 2, and 4 h at 37 °C for *S. aureus* and *S. epidermidis*; (**B**): biofilm formation in each well contained 1 ×, 1/10 ×, and 1/100 × MIC of molecules and an inoculum of 5 × 10^6^ CFU/200 μL, incubated for 24 h at 37 °C for *S. aureus* and *S. epidermidis*. Control bars indicate microorganisms without molecules accepted as 100%. Six wells were used for the tested molecules. Each experiment is representative of two independent tests. All differences between the control and molecule-treated biofilms were statistically significant (**** *p* < 0.0001).

Most bacterial species prefer to live as microcolony communities rather than single cells, since this formation behaves as a shield that keeps away the host immune response and prevents antibiotic effects. The biofilm formation of bacterial cells has highly increased the resistance to antibiotics as well as to host immune responses compared to the planktonic form of life [38,39]. Two important members of *Staphylococci*, in particular *S. epidermidis* and *S. aureus* strains, are responsible for most invasive infectious diseases because of bacterial biofilms [40,41]. For that reason, strong demand for searching the novel lead structures has increased in order to overcome resistance to biofilm-associated infectious diseases [42]. To date, as mentioned in the literature, the quinone scaffolds have shown the ability to disrupt formed bacterial biofilms [38,39] against *S. aureus* [43] and *S. epidermidis* [44]. On these bases, the potential therapeutic application of two highly active quinolinequinones (**QQ2** and **QQ6**) to disrupt bacterial biofilms was analyzed. When we incubated 1/10 MICs of **QQ2** and **QQ6** with MRSA and *S. epidermidis* for adhesion to the wells of microtiter plates, it was observed that neither compound alone inhibited biofilm attachment in relation to incubation time (Figure 4). **QQ2** and **QQ6** showed less than 50% inhibitory activity against MRSA and *S. epidermidis* biofilm formation at 24 h, respectively, based on their concentrations (Figure 4).

## 4. Discussion

The present investigation effectively showed that the quinolinequinones could be considered promising molecules against Gram-positive strains. SAR clearly suggested a correlation between Gram-positive bacteria with the antibacterial profiles. Structural analysis showed that the presence of the ester group is an important moiety for activity. On the other hand, our data also showed that the presence of the ester group at the para position improved the antibacterial activity. In addition, two active quinolinequinones (**QQ2** and **QQ6**) against *S. aureus* were studied for their antimicrobial mechanism via a time-kill kinetic assay and antibiofilm efficacy. *S. aureus* is a significant pathogen in both hospitals and in the community. Methicillin-resistant *S. aureus* isolates are often resistant to other classes of antibiotics (through different mechanisms), making treatment options limited. This has led to the search for new compounds active against these strains [45]. The present study has improved our knowledge of the properties of QQ molecules by showing the extended activity of **QQ2** and **QQ6** against clinically resistant *Staphylococcus* spp. isolates. Additionally, in our study, **QQ6** demonstrated synergistic interaction with levofloxacin against both *S. aureus* and *S. epidermidis* clinically resistant isolates. Synergistic combination therapy applications using existing antibiotics have gained importance, increasing the possibilities of providing active initial therapy, preventing further resistance development, and treating infections by clinically resistant *Staphylococcus* spp. isolates. The compounds **QQ2** and **QQ6** selected for the study of antibiofilm activity among the QQ compounds did not exhibit significant antibiofilm activity. However, to the best of our knowledge, this is the first study to examine the antibiofilm and bactericidal activities of quinolinequinones against clinically resistant strains, which adds special value to this study. In addition, the activity of levofloxacin, when combined with **QQ6**, shows promise as an alternative to treatments for infections caused by clinically resistant *Staphylococcus* spp. As a result, in addition to the promising activity of the combination of levofloxacin and **QQ6** compound against clinically resistant *Staphylococcus* spp. isolates, further clinical studies would be beneficial to determine the clinical effect of this combination and to investigate and develop the efficacy of this regimen in the treatment of infections with clinically resistant *Staphylococcus* spp. isolates.

## Figures and Tables

**Table 1:** In vitro antibacterial activity results from the quinolinequinones (**QQ1**–**9**).

ID	Microorganisms						
	Gram-Negative Bacteria (MIC, μg/mL)				Gram-Positive Bacteria (MIC, μg/mL) ^a^		
	*P. aeruginosa*	*E. coli*	*K. pneumoniae*	*P. mirabilis*	*S. aureus*	*S. epidermidis*	*E. faecalis*
**QQ1**	- ^b^	625	1250	1250	1.22 *(6.84)*	1.22 *(6.84)*	625 *(3503.76)*
**QQ2**	1250	1250	1250	1250	2.44 *(13.69)*	1.22 *(6.84)*	19.53 *(109.49)*
**QQ3**	1250	1250	625	1250	2.44 *(13.69)*	1.22 *(6.84)*	9.76 *(54.75)*
**QQ4**	1250	1250	1250	1250	-	625 *(3371.21)*	1250 *(6742.43)*
**QQ5**	1250	1250	-	1250	1.22 *(6.58)*	1.22 *(6.58)*	19.53 *(105.35)*
**QQ6**	1250	625	625	625	1.22 *(6.58)*	1.22 *(6.58)*	4.88 *(26.34)*
**QQ7**	-	-	-	-	-	156.25 *(784.97)*	-
**QQ8**	625	-	-	-	-	156.25 *(784.97)*	-
**QQ9**	-	-	-	-	1250 *(6279.78)*	1250 *(6279.78)*	-
**Ceftazidime**	2.44 *(8.93)*	-	-	-	-	-	-
**Cefuroxime-Na**	-	4.88 *(21.88)*	4.88 *(21.88)*	2.44 *(10.94)*	1.22 *(5.47)*	-	-
**Cefuroxime**	-	-	-	-	-	9.76 *(46.02)*	-
**Amikacin**	-	-	-	-	-	-	128 *(223.84)*

^a^ = The reported MIC values in parenthesis are presented in µM for each analog. ^b^ = >1250 µg/mL.

**Table 2:** In vitro antifungal activity results from the quinolinequinones (**QQ1**–**9**).

ID	Microorganisms		
	Fungi (MIC, μg/mL) ^a^		
	*C. albicans*	*C. parapsilosis*	*C. tropicalis*
**QQ1**	1250 *(7007.52)*	156.25 *(875.94)*	625 *(3503.76)*
**QQ2**	156.25 *(875.94)*	156.25 *(875.94)*	1250 *(7007.52)*
**QQ3**	1250 *(7007.52)*	156.25 *(875.94)*	625 *(3503.76)*
**QQ4**	1250 *(6742.43)*	625 *(3371.21)*	1250 *(6742.43)*
**QQ5**	1250 *(6742.43)*	78.12 *(421.40)*	625 *(3371.21)*
**QQ6**	1250 *(6742.43)*	156.25 *(842.80)*	625 *(3371.21)*
**QQ7**	4.88 *(24.53)*	4.88 *(24.53)*	- ^b^
**QQ8**	4.88 *(24.53)*	2.44 *(12.27)*	-
**QQ9**	78.12 *(392.49)*	312.50 *(1569.94)*	-
**Clotrimazole**	4.88 *(14.50)*	-	-
**Amphotericin B**	-	0.5 *(0.83)*	1 *(1.35)*

^a^ = The reported MIC values in parenthesis are presented in µM for each analog. ^b^ = >1250 µg/mL.

## Abbreviations

APIs	Active pharmaceutical ingredients
ATCC^®^	American Type Culture Collection
CLSI	Clinical Laboratory Standards Institute
MRSA	Methicillin-resistant *Staphylococcus aureus*
MICs	Minimum inhibitory concentrations
NCCLS	National Committee for Clinical Laboratory Standards
PBMCs	Peripheral blood mononuclear cells
SAR	Structure–activity relationship models
TKC	Time-kill Curve
